# Development and evaluation of a multiplex polymerase chain reaction in real-time for differential diagnosis of *Moraxella*-induced keratoconjunctivitis in livestock

**DOI:** 10.14202/vetworld.2023.2526-2532

**Published:** 2023-12-28

**Authors:** Vitaliy Strochkov, Rano Sattarova, Karlygash Boranbayeva, Flyura Bakiyeva, Kuandyk Shynybayev, Batyrbek Aitzhanov, Markhabat Kassenov

**Affiliations:** 1 Laboratory of Bacteriology, LLP “Kazakh Scientific Research Veterinary Institute”, Almaty 050016, Kazakhstan; 2 Kazakhstan-Japan Innovation Centre, Kazakh National Agrarian Research University, 050010 Almaty, Kazakhstan

**Keywords:** *Moraxella bovis*, *Moraxella bovoculi*, *Moraxella ovis*, *Moraxella* spp, multiplex real-time polymerase chain reaction, Pinkeye

## Abstract

**Background and Aim:**

Infectious bovine keratoconjunctivitis (IBK) is a prevalent ocular disease that affects livestock, leading to substantial economic losses due to reduced production and culling of infected animals. *Moraxella* spp. is common bacterial pathogens that can cause keratoconjunctivitis in livestock. Therefore, rapid and accurate diagnosis is crucial for effective treatment and disease control. This study aimed to develop a multiplex real-time polymerase chain reaction (mRT-PCR) assay for the detection and differentiation of *Moraxella bovoculi*, *Moraxella ovis*, and *Moraxella bovis*.

**Materials and Methods:**

Three reference strains of *Moraxella* as positive controls and 36 lacrimal swab samples collected from cattle were used to evaluate the developed mRT-PCR assay DNA extraction that was performed using the RIBO-sorb DNA/RNA extraction kit. Primers and probes were designed using the SpeciesPrimer pipeline. The annealing temperature, primer and probe concentrations, and sensitivity and specificity of the assay were optimized.

**Results:**

An mRT-PCR assay was developed to detect pathogens associated with IBK in cattle on the basis of optimized parameters. The specificity and sensitivity of this assay were confirmed using samples containing individual pathogens (O – *M. ovis*, B – *M. bovis*, and BO – *M. bovoculi*), combinations of two pathogens (O-B, B-BO, and O-BO), and when the DNA of all three pathogens was present in a single reaction (O-B-BO). The analytical sensitivity of mRT-PCR for detecting *M. ovis* and *M. bovoculi* DNA was 21 copies or 50 fg per reaction, whereas that for *M*. *bovis* was 210 copies or 500 fg per reaction. In addition, this assay has been tested on samples isolated from the affected eyes of cattle in the Akmola region of the Republic of Kazakhstan.

**Conclusion:**

For the first time in the Republic of Kazakhstan, the proposed mRT-PCR assay for the simultaneous detection of three *Moraxella* spp. pathogens has been developed. This assay exhibits the required specificity and high sensitivity for m RT-PCR, facilitating the timely implementation of effective measures for disease control and the prevention of economic losses. These losses are linked to a reduction in livestock breeding value, a reduction in meat and milk production, a reduction in the reproductive performance of heifers, resulting in fewer offspring, as well as costs related to the treatment of affected animals.

## Introduction

Infectious bovine keratoconjunctivitis (IBK), commonly known as “pinkeye,” is a prevalent ocular disease affecting livestock, leading to substantial economic losses due to decreased production and the culling of infected animals. *Moraxella* spp. is recognized bacterial pathogens capable of inducing keratoconjunctivitis (KC) in livestock, emphasizing the critical need for rapid and accurate diagnosis to ensure effective treatment and disease control.

Moraxellosis is the most frequently observed ocular ailment in cattle in Kazakhstan. The susceptibility to this disease is particularly strong in imported beef cattle such as Aberdeen Angus and Hereford. In addition, moraxellosis among certain local breeds, including Auliekolskaya and Kazakh white-headed [1, 2] have been documented. Moraxella KC has a significant economic impact globally, ranging from USD 150 to USD 230 million in some countries such as United States, Australia, New Zealand [3, 4]. Notably, observations underline the vulnerability of calves to this disease and the potential for further dissemination among various breeds in the Republic of Kazakhstan [1, 2].

Infectious KC affects a wide range of animals, including cattle, sheep, goats, camels, pigs, and birds. The sources of infectious agents include sick animals as well as asymptomatic carriers, shedding these microorganisms through conjunctival secretions and nasal mucus. Transmission may occur through direct or indirect contact and mechanical vectors, such as domestic flies, stingers, and field species. The disease is observed throughout the year, but it has a higher prevalence in spring, summer, and autumn. Predisposing factors include suboptimal zoo hygienic conditions, inadequate nutrition, dry and dusty environments, intense ultraviolet radiation, and elevated fly activity during warm seasons [5–7].

Infectious KC is a polyetiological disease, and various causative agents have been identified [8]. At present, based on data from the National Centers for Biotechnology Information, the *Moraxella* genus comprises 22 species [9, 10], with three of these species, *Moraxella bovis*, *Moraxella ovis*, and *Moraxella bovoculi*, identified in cases of IBK [11].

Diagnosing *Moraxella*-associated KC necessitates the application of diverse laboratory techniques, including bacterial culture, polymerase chain reaction (PCR), and serologic assays. Antibiotics, anti-inflammatory drugs, and topical ocular treatments are available for this condition.

Traditional diagnostic modalities, such as bacterial culture and microscopy, are limited in terms of sensitivity, specificity, and expediency. Recently, PCR has emerged as a robust method to detect pathogens in clinical specimens.

This study aimed to develop a multiplex real-time PCR (mRT-PCR) for the differential diagnosis of *Moraxella*-induced IBK in livestock The mPCR assay is uniquely designed to simultaneously detect and differentiate *M. bovis*, *M. bovoculi*, and *M. ovis*, the three primary species associated with KC in livestock. Implementing mRT-PCR in a clinical context can significantly enhance the management of IBK. By providing rapid and accurate identification of the causative pathogens, mPCR can help control the spread of disease and reduce economic losses in the livestock sector.

## Materials and Methods

### Ethical approval

This study was approved at a meeting of the Local Ethical Commission of the Kazakh Research Veterinary Institute of the Science Committee of the Ministry of Education and Science of the Republic of Kazakhstan (Approval no.1 on January 05, 2021).

### Study period and location

This study was conducted from December 2021 to September 2022. This study was conducted in the Laboratory of Bacteriology of the Kazakh Research Veterinary Institute and Green Biotechnology and Cell Engineering of the Kazakh-Japanese Innovation Center at the Kazakh National Agrarian Research University.

### Reference strains

Reference strains of *M. bovis* (American type culture collection [ATCC] 17948), *M. bovoculi* (ATCC BAA-1259), and *M. ovis* (ATCC 33078) were procured from the ATCC located in Manassas, Virginia to evaluate reaction parameters, test specificity, and analytical sensitivity.

### Sample collection

To test the developed mRT-PCR, 35 bovine ocular swab samples were taken from the eyes of clinically ill and asymptomatic animals in three farms in the Akmola region of the Republic of Kazakhstan. Sampling was conducted according to the sampling protocol [12].

### DNA extraction

Bacterial colonies were transferred from the plates using a sterile swab and suspended in 200 μL of sterile water by vigorous stirring for reference strain. The lacrimal swab samples were vigorously stirred in 1 mL liquid Amies medium before extraction. DNA was extracted from 200 μL of the swab suspension using RIBO-sorb DNA/RNA extraction kit (AmpliSense, Russia) according to the manufacturer’s instructions. The concentration and purity of extracted total RNA were determined using NanoDrop 2000 spectrophotometer (Thermo Scientific, USA). Isolated DNA was stored at −20°C until further use.

### Primers and probes selection

Table-1 details the primer and probe sequences employed in the mRT-PCR. These primers and probes was designed using a species primer pipeline [13], which enabled automated high-throughput screening for species-specific target regions and the creation of specialized primers. Fluorescein was labeled at the 3′ end of the probe, and the quencher was attached to the 5′ end.

**Table-1:** Primers and probes of the mRT-PCR assay for the identification of *Moraxella* [13].

Oligonucleotides	Gene	Sequence, 5`-3`
MbovocF-2	g750	CCTTAGTGCCGATCTTGACTGT
MbovocR-2		ACTGTAATTGTTCGCGCATGTC
MbovocP-2		FAM-TCTCTAGCGTGCCTGCACTGTCC-BHQ1
MovF-1	g446	GGGAAATCGCACGGCTAAGA
MovR-1		TGGTCTCGGTTTGGGTTTGT
MovP-1		JOE-CCAGCCTTATATCGCAAAATGACCGCC-BHQ1
MbovF-1	ppiD	GCATGATTGACAGGGCGTTG
MbovR-1		GACAACTGCGTGCGAAACAT
MbovP-1		ROX-ACTGCAAGCTGACCCGACTTTCCA-BHQ2

Mbovoc=*Moraxella bovoculi*, Mov=*Moraxella ovis*, Mbov=*Moraxella bovis*, F=Forward primer, R=Reverse primer, P=Probe. mRT-PCR=Multiplex real-time polymerase chain reaction

### Optimization of PCR conditions

Optimization of the RT-PCR assay involves systematic evaluation of different parameters and concentration ranges. The annealing temperature was varied from 52°C to 62°C. Primers for each target gene were tested within the range of 50–800 nM, and probe concentration was assessed between 150 and 250 nM.

### Analytical sensitivity

A series of 10-fold serial dilutions of genomic DNA derived from reference strains of *Moraxella* were meticulously prepared to assess the sensitivity of the RT-PCR assay. Using genomic DNA as the matrix, these dilutions ranged from 5 to 0.00005 ng/μL.

### Specificity

Genomic DNA from closely related bacterial strains was used to gauge the specificity of the mRT-PCR assay. The specificity analysis included 5 ng of genomic DNA from each of these pathogens.

### Confirmation of mRT-PCR suitability

A total of 35 lacrimal swab samples collected from cattle were examined to verify the suitability of the mRT-PCR assay. The results obtained from the mRT-PCR analysis were further validated using primers and probes acquired from a previous publication as described by Shen *et al*. [14] because commercial kits were not available.

## Results

### Optimization of the RT-PCR assay

Optimization of RT-PCR assay encompasses determination of the optimal annealing temperature and concentration of primers and probes. The optimization process was conducted separately for each pathogen. Within the annealing temperature range of 52–62°C, it was observed that 56°C produced the most stable and early amplification curve. To determine the optimal primer and probe concentrations, various concentrations, spanning from 50 nM to 800 nM for primers and 150 nM to 250 nM for probe, were systematically examined. The optimal primers and probe concentrations for each pathogen are presented in Table-2.

**Table-2:** Optimal conditions of annealing temperature and oligonucleotide concentration for RT-PCR assay.

Species	Annealing temperature of primers, °C	Concentration of forward primers, nM	Concentration of reverse primers, nM	Concentration of probe
*Moraxella ovis*	56	400	200	250
*Moraxella bovis*	56	600	200	300
*Moraxella Bovoculi*	56	600	600	250

RT-PCR=Real-time polymerase chain reaction

In subsequent experiments, mRT-PCR was conducted using primers and probes for all three pathogens in a single reaction. To achieve this, a reaction mixture was prepared in a volume of 20 μL, containing the reaction components at previously determined optimized concentrations: 1× SE buffer for Taq DNA polymerase (60 mM Tris-HCl, 1.5 mM MgCl_2_, 25 mM KCl, 10 mM 2-mercaptoethanol, 0.1% Triton X-100), 0.25 mM dNTPs, 1 mM MgCl_2_, 1 unit of Taq DNA polymerase (SibEnzyme, Russia), and a mixture of primers and probes (MovF-1–400 nM, MovR-1–200 nM, and MovP-1–250 nM; MbovF-1–600 nM, MbovR-1–100 nM, and MbovP-1–300 nM; and MbovocF-2–600 nM, MbovocR-2–600 nM, and MbovocP-2–250 nM). The amplification conditions included an initial step at 95°C for 1 min, followed by 45 cycles of 95°C for 30 s, 56°C for 30 s (with fluorescence detection in the green, orange, and yellow channels), and 72°C for 30 s. StepOne Plus RT-PCR systems (Applied BioSystems, USA) and Rotor-Gene Q Plex RT-PCR systems (Qiagen, Germany) were used for PCR amplification.

These optimized conditions were used for subsequent sensitivity determination, specificity testing, and confirmation of the RT-PCR assay using clinical samples.

### Determination of the sensitivity

**Figure-1:**
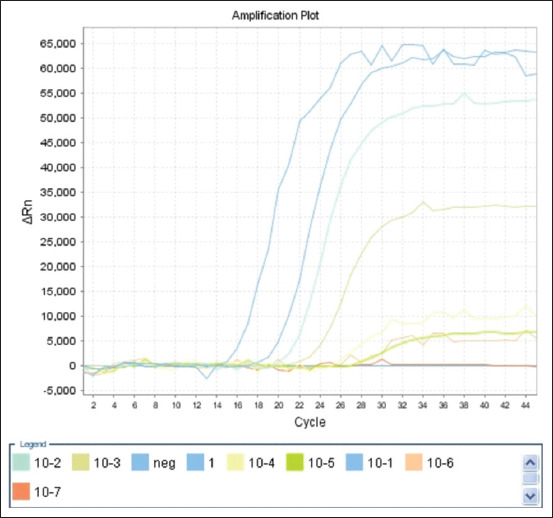
Amplification curves of 10-fold serial dilutions of genomic DNA (5–0.00005 ng/μL per reaction) *Moraxella bovoculi*.

**Figure-2:**
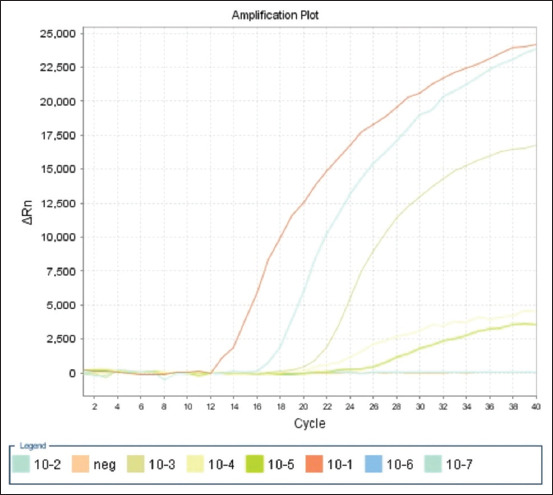
Amplification curves of 10-fold serial dilutions of genomic DNA (5–0.00005 ng/μL per reaction) *Moraxella bovis*.

**Figure-3:**
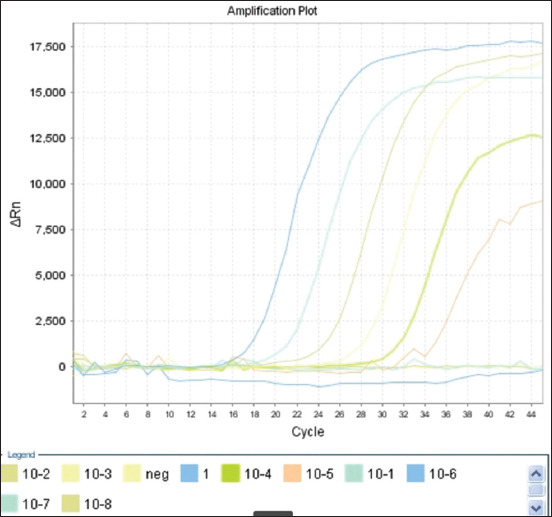
Amplification curves of 10-fold serial dilutions of genomic DNA (5–0.00005 ng/μL per reaction) *Moraxella ovis*.

Analytical sensitivity was determined by testing 10-fold serial dilutions of genomic DNA from reference strains of *Moraxella* spp., ranging from 5 to 0.00005 ng/μL for each reaction. Figures 1–3 show amplification curves for different DNA concentrations. The number of DNA copies obtained at each DNA concentration is presented in Table-3. The analytical sensitivity of mRT-PCR for detecting *M. ovis* and *M. bovoculi* DNA was 21 copies or 50 fg per reaction, whereas that for *M. bovis* was 210 copies or 500 fg per reaction.

**Table-3:** Number of DNA copies of 10-fold serial dilutions of genomic DNA.

Dilution of bacterial DNA	Amount of DNA per reaction, ng	Number of DNA copies
Stock	5	2,105,595
10^-1^	0.5	210,559
10^-2^	0.05	21,056
10^-3^	0.005	2,106
10^-4^	0.0005	210
10^-5^	0.00005	21
10^-6^	0.000005	2
10^-7^	0.0000005	0.2
10^-8^	0.00000005	0.02

### Determination of the specificity

To verify the specificity, three reference strains of *Moraxella* genus were used along with non-target DNA from bacteria, including *Salmonella enterica*, *Streptococcus equi*, *Helicobacter pylori*, and *Pasteurella equi*. Specificity was evaluated using single-plex and mRT-PCR methods.

**Figure-4:**
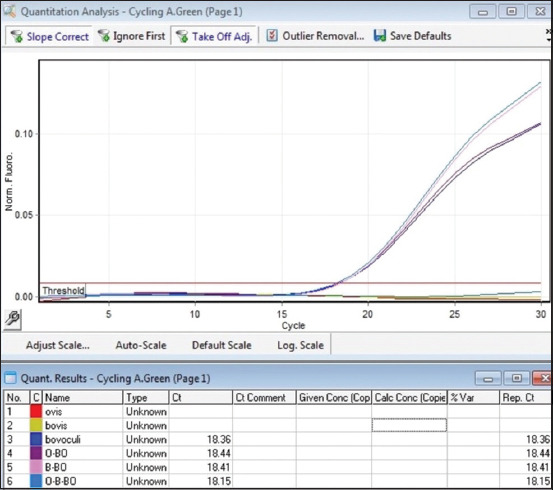
Determination of the specificity of multiplex real-time polymerase chain reaction green channel (*Moraxella bovoculi*).

**Figure-5:**
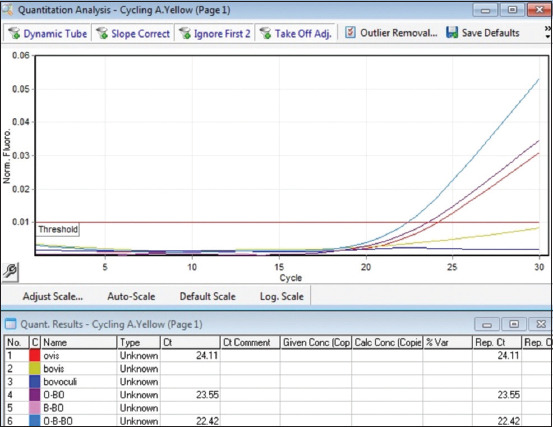
Determination of the specificity of multiplex real-time polymerase chain reaction yellow channel (*Moraxella ovis*).

**Figure-6:**
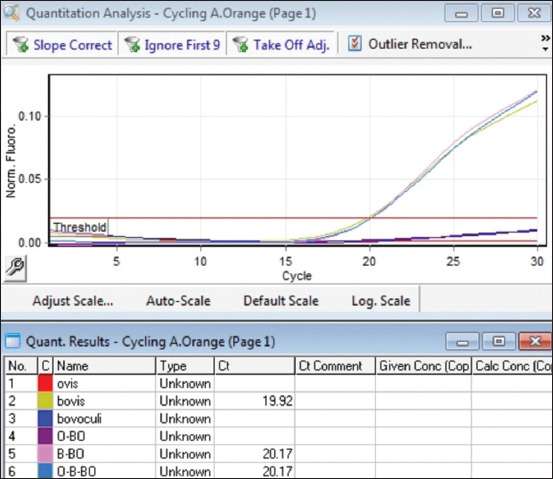
Determination of the specificity of multiplex real-time polymerase chain reaction orange channel (*Moraxella bovis*).

In single-plex PCR, a positive growth of the fluorescence curve was solely evident in the presence of specific DNA from the *Moraxella* genus, with no such signal for other bacterial species. These observations highlight the marked specificity of the primers and samples selected. Samples containing individual pathogens in the reaction, as well as combinations of two or three pathogens in one reaction, were examined to assess multiplex PCR specificity. These results are illustrated in Figures 4–6.

The analysis clearly demonstrated the high specificity of *Moraxella* DNA detection, with no false-positive results.

### Evaluation of developed mRT-PCR assay

The performance of the developed mRT-PCR assay was assessed using lacrimal swab samples collected from clinically affected and asymptomatic animals from three different farms located in the Akmola region of the Republic of Kazakhstan.

Samples were procured from three separate farms designated as farms #1, #2, and #3. Although animals at farms #1 and #2 exhibited clinical symptoms of KC, those at farm #3 were completely asymptomatic. A total of 35 sampled animals participated in the study. The established mRT-PCR protocol was employed to determine the presence of *Moraxella* pathogens in the collected samples. Among the positive *M. bovoculi* cases (n = 15), *M. ovis* was identified in five samples, and *M. bovis* was detected in a single sample. An RT-PCR was performed using the primers proposed by Shen *et al*. [14] to validate the findings.

The comprehensive results are summarized in Table-4 [14]. Collective outcomes affirm the reliability and efficacy of the developed mRT-PCR assay for the precise differential diagnosis of *Moraxella* spp. This assay has successfully demonstrated the ability to accurately identify and differentiate various *Moraxella* pathogens, highlighting its potential value as a robust diagnostic tool.

**Table-4:** Evaluation of the developed RT-PCR assay.

Farm	Number of samples	Results of developed mRT-PCR			Primers proposed by Shen *et al*. [14]		
	
		*MoraxellaBovoculi* (%)	*Moraxella ovis* (%)	*Moraxella bovis* (%)	*Moraxella Bovoculi* (%)	*Moraxella ovis* (%)	*Moraxella bovis* (%)
#1	16	6 (37.5)	1 (6.25)	1 (6.25)	6 (37.5)	1 (6.25)	1 (6.25)
#2	9	9 (100)	4 (44.4)	-	9 (100)	4 (44.4)	-
#3	10	-	-	-	-	-	-
Total	35	15 (42.8)	5 (14.2)	1 (2.8)	15 (42.8)	5 (14.2)	1 (2.8)

mRT-PCR=Multiplex real-time polymerase chain reaction

## Discussion

Monitoring carried out in the Republic of Kazakhstan from 2016 to 2019 revealed the prevalence of eye diseases caused by *Moraxella* spp. across all regions of the country, especially in areas where breeding animals, especially meat-producing breeds, are imported. The incidence rate can reach up to 27% [1].

Several types of pathogenic IBK inflammation in cattle and *Moraxella* are among the most common diseases of the microbiome of the upper respiratory tract and eyes. The pathogenicity of *M. bovis* has been attributed to virulence, cytotoxicity, and reproducibility in laboratory animals. The pathogenesis of IBK has been studied etiological role of *M. bovis*, *M. bovoculi*, and *M. ovis* [15].

Several molecular studies have investigated the 3′ region of the cytotoxin gene in *M. bovis*, *M. bovoculi*, and *M. ovis* [16]. Techniques such as matrix-assisted laser desorption ionization–time of flight have been employed for biomarker determination [17,18], and PCR-Restriction Fragment Length Polymorphism analysis has been utilized for differentiation in IBK [19]. A mRT-PCR protocol has also been developed to detect five pathogens [20].

Geographic variations in *Moraxella* have been explored through whole-genome sequencing [21], and different techniques such as RAPD-PCR, JWP1-JWOPA07-PCR, ERIC-PCR, and sequencing of the 16S-23S intergenic regions have been evaluated [22].

The present study contributes to the existing body of knowledge by presenting a novel approach for the detection and differentiation of *Moraxella* species involved in IBK. While the previous studies have predominantly focused on employing the 16S gene for PCR-based detection [14], our research introduces the innovative use of primers and probes targeting conserved regions across the entire genome of these pathogens. A mRT-PCR assay enables the simultaneous identification of *M. ovis*, *M. bovis*, and *M. bovoculi*, thus providing a comprehensive tool for accurate pathogen differentiation.

In addition, the successful adaptation of the mRT-PCR assay to lacrimal swab specimens offers a practical and non-invasive means of diagnosing and monitoring IBK. This can significantly impact livestock health management and disease control, allowing for early detection, prompt treatment and mitigation of economic losses associated with reduced productivity and potential culling of infected animals.

The results of this study lay the groundwork for further research and practical implementation of the mRT-PCR assay in veterinary healthcare systems, ultimately contributing to improved livestock health and economic outcomes.

## Conclusion

The proposed mRT-PCR method for simultaneous detection of three IBK pathogens has been developed for the first time in the Republic of Kazakhstan. This assay exhibits the required specificity and high sensitivity for real-time PCR, facilitating the timely implementation of effective measures for disease control and the prevention of economic losses. These losses are linked to a reduction in livestock breeding value, a reduction in meat and milk production, a reduction in the reproductive performance of heifers, resulting in fewer offspring, as well as costs related to the treatment of affected animals.

## Authors’ Contributions

VS, RS, MK, and FB: Conceptualization, methodology. VS, KB, KS, and BA: Investigation and formal analysis. VS, RS, and KB: Writing-original draft. VS, MK, and RS: Writing-review and editing. MK and FB: Project administration. All authors have read, reviewed, and approved the final manuscript.
